# Protocol for a first-in-human feasibility study of T regulatory cells (TR004) for inflammatory bowel disease using (ex vivo) Treg expansion (TRIBUTE)

**DOI:** 10.1136/bmjopen-2024-092733

**Published:** 2025-01-23

**Authors:** Beverley Rodger, Jennifer Clough, Joana Vasconcelos, James B Canavan, D Macallan, A Toby Prevost, Graham M Lord, Peter Irving

**Affiliations:** 1 King’s College London Faculty of Life Sciences and Medicine, London, UK; 2 Dept of Primary Care and Public Health Sciences, King’s College London, London, UK; 3 Department of Genitourinary Medicine, St. George’s Healthcare NHS Trust, London, UK; 4 Cicely Saunders Institute, King’s College London, London, UK; 5 MRC Centre for Transplantation, King’s College London, London, UK

**Keywords:** Inflammatory bowel disease, Adult gastroenterology, Gastroenterology, Patient-Centered Care

## Abstract

**Introduction:**

Crohn’s disease (CD) is a chronic, immune-mediated inflammatory bowel disease (IBD), presenting with symptoms of abdominal pain and bleeding from the gastrointestinal tract. There is no known cure. In vitro-expanded ‘thymus-derived’ regulatory T cells (tTreg) have shown promise in preclinical models of IBD, leading to interest in their use as a potential therapy in CD. We present a study protocol for a first-in-human study of Tregs for IBD using ex vivo Treg expansion. This study will explore the preliminary safety and tolerability of a single dose of Treg immunotherapy and will inform the design of a subsequent larger trial.

**Methods and analysis:**

Four patients will be recruited from gastroenterology clinics at Guy’s and St Thomas’ NHS Foundation Trust. Eligible participants are those who are at least 18 years old, have a diagnosis of active moderate to severe CD and have failed to respond to or tolerate at least two prior lines of standard medication. Participants receive a single dose of autologous ex vivo-expanded Tregs and will be followed up to week 21 to collect safety and exploratory efficacy data. Additional safety monitoring will occur at 1 and 2 years post-dose. The primary endpoint is defined as the occurrence of dose-limiting toxicity occurring within 5 weeks post-infusion.

**Ethics and dissemination:**

The study protocol and related documents have been approved by a NHS Research Ethics Committee, the Health Research Authority and the Medicines and Healthcare products Regulatory Agency for Clinical Trial Authorisation. It is intended that the results of the trial will be presented at international conferences and will be submitted for publication in a peer-reviewed scientific journal.

**Trial registration number:**

NCT03185000

**STRENGTHS AND LIMITATIONS OF THIS STUDY**

- First-in-human study of a novel, gut-targeted cell product to treat patients with Crohn’s disease.
- Novel investigational medicinal product manufacturing method.
- Thorough safety precautions/analysis for treated participants.
- Small sample size limits ability to assess safety and efficacy.
- Potential to improve technical aspects of manufacturing process.

## Introduction

### Background and rationale

 Crohn’s disease (CD) is a chronic immune-mediated inflammatory bowel disease (IBD), characterised by transmural inflammation of the gastrointestinal (GI) tract. It is typically diagnosed in early adulthood and can cause significant morbidity, with symptoms of abdominal pain, change in bowel habit and bleeding from the GI tract, and may be complicated by intestinal strictures or fistulae. There remains an unmet need to develop novel therapies for CD, as current drug treatments frequently fail to maintain long-term remission and may be complicated by significant side effects. Currently available CD therapies, including corticosteroids, Janus kinase (JAK) inhibitors, anti-Tumor necrosis factor (anti-TNF), anti-integrin and anti-Interleukin-12/23R (anti-IL-12/23R) therapy, seek to reduce immune activation in the gut by targeting effector immune mechanisms.

#### Evidence for the role of regulatory T cells in Crohn’s disease pathogenesis

The maintenance or loss of intestinal homeostasis hinges on the balance between inflammatory effector T cells (Teff), which have been implicated in auto-immunity and transplant rejection, and a population of immunoregulatory T cells (Treg).^1^,^3^

Tregs are a unique subset of CD4+ T cells with powerful immunosuppressive action. They are defined by expression of the master transcriptional regulator FOXP3 and high constitutive expression of IL-2RA (CD25), the high-affinity receptor for IL-2.^4^,^6^ Tregs are capable of exerting tolerising immune responses through several mechanisms, including direct cell-cell contact and secreted products. The expression of inhibitory cytokines by Tregs, including IL-10, Transforming growth factor beta (TGFβ) and IL-35, has been implicated in regulatory function.^7^ IL-10 can suppress the ability of antigen-presenting cells (APC) to stimulate T cells by inhibiting APC maturation and expression of costimulatory molecules, and by suppression of cytokine production.^8^ IL-2 is required for Teff activity. Tregs can decrease interstitial IL-2 and inhibit IL-2 synthesis by Teffs.^9 10^ Tregs also express the inhibitory molecule CTLA-4, by which they can exert control over effector cell function.^11^

Lamina propria (LP) Tregs are increased in the mucosa and decreased in the blood of patients with active CD, compared with healthy controls.^12^,^14^ LP Tregs taken from the inflamed CD mucosa suppress proliferation of conventional CD4+CD25lo/int Teffs obtained from blood but not from the LP, suggesting that mucosal Teff in active CD may be resistant to Treg-mediated suppression.^15^ However, our group and others have shown that in vitro-expanded Tregs generated from blood in the presence of rapamycin are more potently suppressive than freshly isolated Tregs.^16 17^ Thus, in vitro-expanded Tregs from blood may overcome mucosal Teff resistance to suppression. Consequently, parenteral therapy with autologous ex vivo-expanded Tregs generated from Crohn’s blood may be an attractive approach to address defects in, or resistance to, mucosal Treg function in CD. We, and others, have shown that Tregs can be infused into animals with IBD, resulting in prevention or reversal of colitis^18^,^22^ Further, expanded human Tregs promote skin transplant tolerance, prevent transplant arteriosclerosis, and graft versus host disease (GvHD), in immunodeficient mice reconstituted with human peripheral blood mononuclear cells.^23^,^25^ It has also been shown that Tregs expanded in vitro from umbilical cord blood (UCB) are safe and efficacious at preventing GvHD following UCB transplantation.^26^ Other recent phase I studies demonstrate that adoptively transferred Tregs are safe in the context of haematopoietic stem cell transplantation, GvHD, and type 1 diabetes, while providing an early signal of efficacy.^1 27 28^

#### Manufacturing an ex vivo-expanded Treg product

We and others have optimised the conditions for in vitro expansion of Tregs from peripheral blood (PB).^6 29 30^ Human Tregs isolated from PB or inflamed CD mucosa contain a subpopulation of IL-17+ effector T cells.^31^ In addition, human Tregs can be induced to express IL-17 or IFNγ in vitro.^32^ IL-17 is a proinflammatory cytokine implicated in the development of inflammatory disorders including CD.^33^ Adoptive transfer of expanded cells with the potential to express effector cytokines and contribute to CD pathogenesis could be deleterious. Our previous work has identified that the starting population for Treg expansion from the PB has a critical effect on the phenotype of the expanded cell population.^30 34^ Tregs from a highly pure FACS-sorted ‘naïve’ CD4+CD25hiCD127loCD45RA+ precursor population demonstrated enhanced suppressive ability and reduced Th17 plasticity in vitro compared with a FACS-sorted CD4+CD25hiCD127loCD45RA- or MACS-enriched CD8-CD25+ population.

The inclusion of rapamycin in culture during the Treg expansion process also prevents the expansion of Th17 cells, even when expanded Tregs are subsequently exposed to a Th17-favourable environment.^29^,^3135^ Rapamycin-expanded Tregs maintain their in vitro regulatory phenotype after transfer into Nonobese diabetic/severe combined immunodeficient (NOD-SCID) mice despite being exposed to an irradiation-induced proinflammatory environment. No additional rapamycin treatment either in vitro or in vivo is required to maintain their regulatory phenotype.^31^ Rapamycin added to cell culture appears to imprint a fixed CD4+CD25hi phenotype to cells expanded from a ‘naïve’ CD45RA+ population, as evidenced by retention of demethylation at the Forkhead box P3 (Foxp3) locus.

The expression of theheterodimeric integrin α4β7 confers Treg gut-homing ability, through interaction with its ligand mucosal vascular addressin cell adhesion molecule 1 .^36^ Inclusion of all-trans-retinoic acid (ATRA) in Treg expansion media induces expression of α4β7 integrin on the Treg surface. Tregs expanded in ATRA alone retain the ability to secrete IL-17, but Tregs cultured in media including both rapamycin and ATRA demonstrated no IL-17 production.^29^ The combination of rapamycin with ATRA provides an approach for large-scale expansion of functionally potent and phenotypically stable Tregs. We have shown that adoptive transfer of expanded Tregs treated with a highly-specific retinoic receptor α (RARα) receptor agonist leads to improved Treg gut trafficking into human intestinal xenografts in mice.^30 34^ The RARα agonist-treated cell product forms the basis of TR004.

In order to map the fate of infused cells, Tregs are ‘tagged’ through inclusion of deuterated [6,6-2 H2] glucose in the culture media during the expansion process.^27^ This also enables confirmation that infused Treg does not convert to Teff in vivo post infusion. Incorporation of deuterium into the deoxyribose moiety of newly synthesised DNA enables infused Treg and their progeny to be distinguished from pre-existing or endogenously synthesised Treg; their different genomic DNA isotopic profiles can be readily discriminated by mass spectrometry.^37^ Previous clinical studies in human participants with diabetes using a similar approach have demonstrated detectable transferred Tregs in peripheral blood up to 1 year post infusion and shown no detectable Treg to Teff conversion post infusion.^27^ We will use deuterium enrichment added ex vivo during the expansion phase to permit tracking of transferred Tregs to the gut and to determine longevity of the infused Tregs that comprise the cell product (TR004).

This clinical study will thus use autologous, ex vivo-expanded Tregs as a parenteral cell-based therapy to augment regulatory immune responses in the gut of patients with CD.

### Objectives

To explore the preliminary safety and tolerability of a single dose of TR004 in patients with moderate to severe CD who are refractory or intolerant to standard treatment. Further, this trial will inform the design of a subsequent larger trial.

Feasibility objectives: To pilot the operation of the clinical protocol and inform the design of a subsequent larger trial.

- To assess the feasibility of manufacturing the investigational medicinal product (IMP) at the dose selected for this study.
- To assess the feasibility of recruiting to time and target.
- To assess the feasibility of retaining participants for the duration of the study and completion of study visits and assessments.
- To understand the experience of the participants, trial team and data safety monitoring board (DSMB) members, exploring any barriers and challenges to trial performance, recruitment and retention.
- To measure the in vivo lifespan of infused cells, evaluate localisation to gut and ensure no Treg to Teff conversion.

## Methods: participants, interventions and outcomes

### Trial design

This study is an open-label, first-in-human feasibility study of a single dose of TR004 in patients with moderate to severe CD. Four participants will receive a single dose of TR004. Participants will be dosed singly. Safety data will be collected for 5 weeks post administration and reviewed by the DSMB before proceeding to dose the next participant. All participants will be followed up to week 21 to collect further safety and exploratory efficacy data, with additional safety monitoring at 1 and 2 years post dose.

### Study setting

All study activities will occur at Guy’s Hospital, London. TR004 will be manufactured under Good Manufacturing Practice (GMP) conditions at Guy’s Hospital. Patients will be dosed in the Clinical Research Facility (CRF), 15th Floor, Tower Wing, Guy’s Hospital, London.

### Patient and public involvement

A survey was completed by members of a CD patient and public involvement (PPI) group to find out how people with the condition may feel about taking part in this study, including their perspectives on the novel therapy, trial design, and the number and type of assessments involved. This information was written up as a report with recommendations which were incorporated into the patient pathway and patient documentation. In addition, The Patient Information Sheet was reviewed by members of the PPI group.

### Eligibility criteria

#### Inclusion criteria

1. Able and willing to provide written informed consent and able to comply with the protocol requirements.
2. Men or women aged between 18 and 80 (inclusive) years of age at date of consent.
3. A diagnosis of CD established ≥12 weeks prior to date of consent by standard clinical, radiological, endoscopic and histological criteria.
4. Documented moderate to severe CD with a Crohn’s Disease Activity Index (CDAI) ≥220 within 3 months of date of consent.
5. Active CD (mucosal inflammation) including ulceration, as assessed by colonoscopy at screening.
6. Failure to tolerate or to respond, or lost response to at least two prior lines of standard CD medication intended to induce or maintain remission, as determined by the referring gastroenterologist.
7. Stable doses of concomitant medications.

#### Exclusion criteria

1. A diagnosis of ulcerative colitis or IBD-unclassified.
2. CD treatment-naïve patients, defined as patients who have never received or have declined standard CD treatment.
3. History of clinically significant drug or alcohol abuse in the last 12 months prior to date of consent.
4. Any history of primary immune deficiency.
5. Patients with a history of pulmonary embolism or deep vein thrombosis. Current or recent history (within 1 year prior to screening) of major organ or system failure or condition, acute or chronic that in the opinion of the investigator should preclude enrolment, except CD.
6. History of intestinal resection or intra-abdominal surgery within 6 months prior to date of consent.
7. Requirement for immediate or imminent surgical, endoscopic or radiological intervention for indications including (but not limited to) toxic megacolon, obstruction, massive haemorrhage, perforation, sepsis, or intra-abdominal or perianal abscess.
8. Patients with ileostomy or colostomy.
9. Patients with short bowel syndrome (less than 1.5 m of small bowel).
10. Complication of CD such as strictures/stenosis, penetrating disease or any other condition that might require GI surgery.
11. Patients receiving therapeutic enema or suppository, other than required for endoscopy, within 14 days prior to date of consent and/or during the screening period.
12. Patients who are currently using anticoagulants.
13. Use of corticosteroids on the day of leukapheresis sampling, prior to the procedure.
14. Current medically significant infection(s) requiring treatment with intravenous (IV) anti-infectives within 30 days prior to consent or oral anti-infectives for non-CD-related infections within 14 days prior to consent.
15. Participant with an active systemic viral infection.
16. History of tuberculosis.
17. History of severe congestive heart failure (New York Heart Association (NYHA) class III or IV), recent cerebrovascular accident (within 6 months of screening) and any other condition which, in the opinion of the investigator, would put the patient at risk by participation in the study.
18. Patient with a previous history (within 12 months of consent) of dysplasia of the GI tract, or found to have dysplasia during the screening endoscopy
19. Significant laboratory abnormalities, assessed on day 1 at week 0: Haemoglobin (Hb) <100 g/L or White blood cell count (WBC) <3.5 × 10^9^/L or Platelets count (Plt) <100 × 10^9^/L; creatinine >1.5× Upper limit of normal (ULN); total bilirubin >34 µmol/L or (Alanine aminotransferase) ALT >2× ULN or (Gamma-glutamyl transferase) GGT >2× ULN. Elevated unconjugated bilirubin related to Gilbert’s syndrome is allowed.
20. Anti-TNF or ustekinumab therapy within 8 weeks of dosing (day 0). Vedolizumab therapy within five half-lives (15 weeks) of dosing. Exposure to cyclosporine or tacrolimus within 2 weeks of consent.
21. Patient currently receiving total parenteral nutrition (TPN) or planning to receive TPN at any time during the course of the study.
22. Received another investigational drug within 60 days of anticipated study date of consent or five half-lives whichever is greater.
23. Patient who previously received stem cell transplantation.
24. Current evidence of dysplasia or history of malignancy within the last 5 years of consent (except non-melanoma skin cancer, successfully treated squamous cell or basal cell carcinoma, without metastases or localised carcinoma in situ of the cervix).
25. Pregnant and lactating patients.
26. Female patients of childbearing potential and male patients who are not willing to use a highly effective method of contraception for the duration of the trial (defined as consent to W21 visit) to prevent pregnancy, or abstain from heterosexual activity.
27. Allergy to any component/excipients used for the manufacture of TR004.
28. Patient is considered by the Principal Investigator (PI), for any reason, to be an unsuitable candidate for the study.

### Who will take informed consent?

It is the responsibility of the PI or delegate at physician level in line with Guy’s and St Thomas’ Trust (GSTT) consent policy to obtain written informed consent for each patient prior to performing any trial-related procedures.

### Additional consent provisions for collection and use of participant data and biological specimens

Consent will also be obtained for the retention and use of patient samples in related future research.

## Interventions

### Explanation for the choice of comparators

N/A as there is no comparator in this study.

### Intervention description

Eligible patients will receive a single dose of TR004 at 2.5–5.0 × 10^6^ TR004/kg. The TR004 IMP will be thawed by the GMP team, and the thawed 50 mL (2.5–5.0 × 10^6^ TR004/kg) IMP bag will be spiked to a giving set for infusion.

### Criteria for discontinuing or modifying allocated interventions

If the required dose cannot be manufactured, the patient may have to be dosed at a lower dose than the dose originally planned. There may even be instances where the patient cannot be dosed if TR004 manufacture fails. In such a situation, the PI will decide whether it is appropriate to rescreen the patient and repeat the leukapheresis. The patient’s consent to be rescreened would have to be sought. In such instances, leukapheresis can be performed on a maximum of two occasions, at least 6 weeks apart.

### Strategies to improve adherence to interventions

N/A as the trial involves only one infusion of therapeutic product.

### Relevant concomitant care permitted or prohibited during the trial

Data will be collected relating to concomitant medications at every visit. The following concomitant medications will be permitted:

- Azathioprine, mercaptopurine, tioguanine and methotrexate will be permitted as long as the patient has been on treatment for at least 12 weeks prior to dosing, including 8 weeks at a stable dose.
- Corticosteroid treatment will be permitted from 2 weeks prior to dosing (week −2) to week 16 but the dose must be tapered down to 20 mg of prednisolone (or equivalent) 2 weeks prior to dosing. Any alterations during the study must be reviewed by the Chief Investigator (CI), and the dose must not exceed 20 mg of prednisolone (or equivalent). Corticosteroid treatment cannot be initiated after week −2.
- 5-aminosalicylic acid (5-ASA) medications will be permitted but the dose must be stable 2 weeks prior to dosing.
- Antibiotics for the treatment of CD will be permitted but the dose must be stable 2 weeks prior to dosing.

### Provisions for post-trial care

All trial participants will be patients registered with Guy’s and St Thomas’ NHS Trust for provision of their IBD care. Post-trial care will, therefore, be in continuity with care received during the trial. Should clinical review suggest worsening active CD post-trial, step-up medication will be offered according to standard of care.

### Outcomes

#### Primary clinical endpoint

- Rate of dose-limiting toxicities (DLTs) occurring within 5 weeks post infusion of TR004.

Feasibility endpoint

- Amount of TR0004 manufactured per patient.
- Number of participants recruited within the duration of the trial.
- Number of study visits completed.
- Responses to items in questionnaires or surveys exploring the experience of the participants, trial team and DSMB members. This will not form part of the final trial report. Qualitative data collection and any data analysis will be separate from the trial.

#### Secondary endpoints

- Assessment of clinical response:
  - Disease Activity Score (CDAI/PRO-2).
  - Biomarkers analysis (CRP, FCP).
  - Mucosal healing response (SES-CD).
- Assessment of immunological response—in blood and intestinal LP:
  - Numbers and functions of Tregs.
  - Measurement of deuterium-enriched cells.
  - Cytokine levels.
  - Comparison of circulating and localised cells to determine differences and similarities.
  - Description of non-DLT adverse events and those occurring beyond week 5.

### Participant timeline

Trial participants will be reviewed and assessed throughout the trial from Screening to Week 104. Clinical blood tests will be performed throughout the duration of the study, and blood samples for translation research will be collected up to week 21 post infusion. Regular visits will be scheduled up to week 8, with follow ups scheduled on week 16 and week 21 thereafter. Please refer to online supplemental tables 1A,B section for more details.

### Sample size

A sample size of four participants will be recruited to assess the safety and tolerability of TR004 for further investigation in a larger clinical trial. The initial plan was to recruit 24 subjects but a change in funding because of the COVID-19 pandemic required a reduction in sample size and a change to a feasibility study. The sample size was a balance between the available funding and an ability to show feasibility of recruitment and the manufacturing process.

In situations where a patient has to be withdrawn or decides to withdraw from the study, an additional patient will be recruited and dosed if:

- They withdraw prior to the start of their 5-week active period.
- They withdraw during their 5-week active period, and have not yet experienced a DLT.

### Recruitment

Suitable patients for the clinical trial will be identified from gastroenterology clinics at GSTT by the direct care team. The participants’ medical history and results will be reviewed by the clinical team at GSTT to confirm potential eligibility for participation in the study before the Patient Information Sheet (PIS) is given. A prescreening log will be used to record the number of participants potentially eligible but not entered into the trial in order to fulfil Consolidated Standards of Reporting Trials reporting guidelines. A Screening and Enrolment Log and Study Identification (ID) Log will be maintained by the site. Potentially eligible participants that decline to take part will be asked if they are willing to provide a reason, which will be captured anonymously on the prescreening log.

## Assignment of interventions: allocation

### Sequence generation

N/A – —treatment is unblinded and all participants receive intervention.

### Concealment mechanism

N/A—treatment is unblinded and all participants receive intervention.

### Implementation

Only the direct care team can identify and approach potential participants without prior consent (whether at GSTT or their local site). Potential participants will be asked to provide verbal consent for a member of the research team to contact them to discuss the study further.

## Assignment of interventions: blinding

### Who will be blinded

Treatment in this study is unblinded.

### Procedure for unblinding if needed

N/A—treatment in this study is unblinded.

## Data collection and management

### Plans for assessment and collection of outcomes

A TRIBUTE Data Management Plan has been developed to outline the principal procedures and processes used to ensure consistent and efficient collection and management of all data gathered in the study. The electronic Case Report Form (eCRF) (on MedSciNet) has been designed and produced by members of the study research team and incorporates all specifications required by the study protocol. To qualify for the study data entry, all users have been trained in line with the MedSciNet Database User Guideline. The eCRF platform automatically creates a protected audit trail for all data entries and changes. Amendments to eCRF data are recorded in the audit trail with a time and date stamp, along with a user-specified reason for the implemented change.

Source documentation for the study includes (but is not limited to):

- Informed Consent Forms.
- Medical records.
- Clinical reports.
- Laboratory reports.
- Hospital correspondence.
- Patient questionnaires.
- Source Data Workbooks.

The CI will act as custodian for the trial data. They have oversight of the data management processes and from time to time may delegate (where appropriate) any ad hoc data management activities. Ongoing and final data quality checks are/will be performed by Clinical Data Manager, Clinical Trials Manager and Clinical Research Associate before the (eCRF) database is locked.

### Plans to promote participant retention and complete follow-up

Participants will be provided with contact details of the trial study team and will be encouraged to contact the team with any queries or concerns.

Participants who wish to withdraw from trial medication (IMP) will be asked to confirm whether they are still willing to attend the safety follow-up visits as per protocol for the duration of the trial. If participants are not willing to do this, consent will be sought for the trial team to access routine data collected from hospital/General Practitioner (GP) visits for the duration of the safety follow-up period.

### Data management

A specific data management plan has been created for the trial. Data will be entered in the eCRF platform to automatically create a protected audit trail for all data entries and changes.

Access to the trial eCRF platform will be password protected and electronic login credentials will be issued only to authorised individuals.

### Confidentiality

To protect the privacy and identity of trial patients, patients will be assigned a unique patient trial identifier upon enrolment. Only investigators and authorised staff at the trial site will be in possession of documents that link patient names to patient trial identifiers (ie, Informed Consent Form (ICF) and Patient Identification Log).

### Plans for collection, laboratory evaluation and storage of biological specimens for genetic or molecular analysis in this trial/future use

Populations of circulating lymphocytes in the blood of patients undergoing TR004 immunotherapy will be assessed for their phenotype and the production of cytokines including IFNγ, Tumour Necrosis Factor alpha (TNFα), IL-5, IL-10, IL-12, IL-13, IL-17, IL-18, IL-22, IL-23, IL-33, Granulocyte-macrophage colony-stimulating factor (GM-CSF) and oncostatin.

Peripheral blood will also be collected at various study visits to assess the levels of circulating regulatory T cells labelled with deuterium. Please refer to online supplemental table 2 section for more details.

24 punch biopsies will be taken from a combination of the ileum and the colon from each patient undergoing TR004 immunotherapy at screening and at week 8. Lymphocytes localised in the ileum and colon of patients undergoing TR004 immunotherapy will be assessed, and their phenotype and cytokine production will be compared with circulating lymphocytes to determine any differences or similarities between circulating and localised cells. Research stool samples collected during the trial will be sent for microbiome analysis after the end of the study.

## Statistical methods

### Statistical methods for primary and secondary outcomes

In view of the feasibility trial design and the numbers of patients, the statistical analysis approach will be a descriptive presentation of each patient’s data rather than the calculation of summary statistics.

### Interim analyses

Given this is a small feasibility study including a small cohort of only four patients, no formal interim analyses are planned. The study may be terminated prematurely by the sponsor, CI or regulatory authority on the basis of new safety information or for other reasons given by the DSMB, regulatory authority or ethics committee concerned. In the event of premature termination, the sponsor will notify the regulatory authorities within 15 days by providing a detailed written explanation. The CI will inform the research ethics committee (REC). The affected trial participants will also be informed promptly and appropriate follow-up visits will be arranged. No further participant data will be collected.

The clinical trial may be prematurely terminated for the following reasons:

- Serious and/or persistent non-compliance with trial protocol.
- Non-compliance with ethical standards, regulatory requirements or GCP compliance.
- Findings uncovered during monitoring visits, audits or inspections that compromise patient safety or suitability of the site to act as a trial centre.
- Recommendation from DSMB.
- Failure to meet recruitment targets.

During the course of the study, any of the following events will trigger a pause in patient recruitment and an emergency review by the DSMB:

- Death.
- DLT observed in two patients out of four.

In the event of emergency review by the DSMB, a substantial amendment will be submitted to the Medicines and Healthcare products Regulatory Agency (MHRA) for approval to restart the study.

### Methods for additional analyses (eg, subgroup analyses)

Not applicable as this is a small trial with only four patients, there will be no subgroups.

### Methods in analysis to handle protocol non-adherence and any statistical methods to handle missing data

Not applicable as the treatment will be given as a single dose, so protocol non-adherence will not be an issue.

### Plans to give access to the full protocol, participant-level data and statistical code

The full protocol and participant-level dataset will be available on request.

## Oversight and monitoring

### Composition of the coordinating centre and trial steering committee

The Trial Management Group (TMG) is led by Professor Peter Irving, the CI for this study. The group will include the CI, statisticians, clinical trial manager, project manager and representatives of the other teams involved in the delivery of the trial, including the GMP unit, laboratories, data management and the CRF. The TMG will be responsible for the day-to-day management of the trial activities and will meet on a regular basis to discuss any trial-related activities or issues.

The Trial Steering Committee (TSC) will provide advice for the conduct of the trial. It will comprise an independent chair and at least two other members. The TSC members will meet on an ad hoc basis to discuss trial status, recruitment progress and any other relevant issues, and provide recommendations to the TMG/sponsor.

### Composition of the data monitoring committee, its role and reporting structure

A DSMB, which is independent from the sponsor, will be constituted prior to study opening. The DSMB charter will detail membership and terms of reference. The DSMB members will be independent and supported by the trial statistician. In order to ensure patient safety throughout the conduct of the trial, the DSMB will review and evaluate accumulated safety data, study conduct and progress. The DSMB will make the decisions about the continuation, modification or termination of the study.

The DSMB members will convene at defined time points stated in the DSMB charter.

A DSMB meeting would also be triggered if specific safety events occurred (see below).

The DSMB meeting will take place:

- If a DLT is observed in two patients out of four.
- If death of a patient infused with TR004 occurs.
- Or at any other time deemed necessary by the DSMB chair.

### Adverse event reporting and harms

Adverse events (AEs) will be recorded and graded according to the Common Terminology Criteria for Adverse Events.^38^ AEs (including serious adverse events) will be recorded from study entry (once the patient has consented) to week 21 visit. During the safety follow-up period (from end week 21 to week 104), only Suspected Unexpected Serious Adverse Reactions (SUSARs) will be reported.

The delivery of the co-sponsors’ responsibility for pharmacovigilance (as defined in Regulation 5 of the Medicines for Human Use (Clinical Trials) Regulations 2004) has been delegated to the King’s Health Partners Clinical Trials Office (KHP-CTO). The KHP-CTO will report SUSARs to the regulatory authority, the MHRA. The CI and KHP-CTO (on behalf of the co-sponsors) will submit a Development Safety Update Report relating to this trial IMP to the MHRA and REC annually. The CI will submit annually to the main REC an Annual Progress Report.

### Frequency and plans for auditing trial conduct

Data validation checks provide the first quality control step, immediately after an eCRF page is saved as complete. These checks ensure the completeness, plausibility and consistency of manually entered trial data. Programmed edit checks run online at the time of saving each screen/page. Source data verification (SDV) occurs after the automatic validation checks. It is performed by the Clinical Research Associate who visits the trial centre during site monitoring visits. SDV ensures the accuracy of manually entered trial data by comparing eCRF entries against the source data available at the site.

### Plans for communicating important protocol amendments to relevant parties (eg, trial participants, ethical committees)

All protocol amendments are sent for initial review to the sponsor and Research & Development (R&D) Department. Following submission to the relevant regulatory bodies (HRA, MHRA and/or REC depending on the content of the amendment) for review and consequent regulatory body approval, the amendment and approvals are then forwarded on to the R&D Department for confirmation of continued capacity and capability. The local clinical team is then notified that the amendment is ready to be implemented at the centre (and given the instructions about the implementation process and provided with any supplementary training if required). For any amendments to patient-facing literature, the new versions of these documents are presented to the participants at the next possible clinic visit where they can be counselled and informed of the changes of the study and also be reconsented using the new patient-facing documents (if required).

### Dissemination plans

It is intended that the results of the trial will be submitted for publication in a peer-reviewed scientific journal. Results will also be reported and disseminated at international conferences. This trial is subject to an external communications strategy which makes patients and healthcare providers aware of the study and to encourage recruitment. We have partnered with patient groups (eg, Crohn’s & Colitis UK) and GI societies (eg, British Society of Gastroenterology) and have direct communication with lead clinicians in research-active clinical gastroenterology departments.

## Trial status

Trial protocol V.4.0, 10 October 2023.

Start of inclusion: 8 August 2022.

Estimated primary completion: 31 March 2024.

Estimated study completion: 30 June 2025.

Please refer to the Administrative Information part of the online supplemental information section for more administrative details.

## Ethics approval and consent to participate

This protocol and related documents have been submitted for review to a NHS Research Ethics Committee (REC), the Health Research Authority (HRA) and the Medicines and Healthcare products Regulatory Agency (MHRA) for Clinical Trial Authorisation. REC approval was acquired on 12-MAY-2022 (REC Reference: 22/NE/0062) from the North East - York Research Ethics Committee. The Chief Investigator will submit a final report at conclusion of the trial to the KHP-CTO (on behalf of the co-sponsors) and the REC within the timelines defined in the Regulations. The KHP-CTO or delegate will upload the final report to a publicly registered database on behalf of the co-sponsors.

## supplementary material

10.1136/bmjopen-2024-092733 online supplemental file 1

10.1136/bmjopen-2024-092733 online supplemental file 2
