# Supplementary material for: Protocol for a first-in-human feasibility study of T regulatory cells (TR004) for inflammatory bowel disease using (ex vivo) Treg expansion (TRIBUTE)
Source: BMJ Open. 2025 Jan 23;15(1):e092733. doi: 10.1136/bmjopen-2024-092733 (PMC11759877; doi:10.1136/bmjopen-2024-092733)
Supplement: online supplemental file 1 [file bmjopen-15-1-s001.docx]

# Administrative information

**Title.**

Protocol for a first in human feasibility study of T regulatory cells (TR004) for Inflammatory Bowel disease using (ex vivo) Treg expansion (TRIBUTE)

**Trial registration.**

NCT03185000

**Protocol version.**

10 October 2023. Protocol Version 4.0

**Funding.**

Medical Research Council (MRC) by means of a research grant awarded by the MRC Industry Collaboration Agreement (MICA) grant (Grant Reference: MR/N006445/1). Further funding has been provided by the National Institutes of Health and Care Research (via a Clinical Trial Grant and as part of the Biomedical Research Centre and Clinical Research Facility at Guy’s and St. Thomas NHS Foundation Trust and King’s College London).

**Author details.**

Graham Lord (Chief Scientific Investigator): Faculty of Biology, Medicine and Health, University of Manchester, UK

Graham.Lord@kcl.ac.uk

Derek Macallan (Co-Investigator): Institute for Infection & Immunity, St George’s, University of London, UK; Infection Clinical Academic Group, St George’s University Hospitals NHS Foundation Trust, London, UK

macallan@sgul.ac.uk

Toby Prevost (Lead Statistician): Nightingale-Saunders Clinical Trials and Epidemiology Unit, King’s College London, UK

toby.prevost@kcl.ac.uk

Joana C. Vasconcelos (Trial Statistician): Nightingale-Saunders Clinical Trials and Epidemiology Unit, King’s College London, UK

joana.vasconcelos@kcl.ac.uk

James B. Canavan (Research Associate): Peter Gorer Department of Immunobiology, King’s College London, UK

Peter Irving (Chief/Principal Investigator): IBD Unit, Guy’s and St Thomas’ Hospital NHS Foundation Trust, London UK; Peter Gorer Department of Immunobiology, King’s College London, UK

Peter.Irving@gstt.nhs.uk

Jennie Clough (Research Associate): Peter Gorer Department of Immunobiology, King’s College London, UK

jennifer.clough@nhs.net

Beverley Rodger (Research Associate): Peter Gorer Department of Immunobiology, King’s College London, UK

beverley.1.rodger@kcl.ac.uk

(Corresponding author: Peter Irving. IBD Unit, Guy’s and St Thomas’ Hospital NHS Foundation Trust & King’s College London, London, UK. Email: peter.irving@kcl.ac.uk]

**Name and contact information for the trial sponsor.**

Ann Marie Murtagh

King’s Health Partners Clinical Trials Office

M: +44 020 71885732

E: QM.KHPCTO@kcl.ac.uk

**Role of sponsor.**

The Sponsor is responsible for the planning, initiation and management of the study. This includes monitoring and supervising the progress of the trial towards its overall objectives, including reviewing recruitment rates, adherence to the protocol and analysing the results of the trial.

A key part of the Sponsor’s obligations includes ensuring that the rights, safety and wellbeing of the trial participants are the most important considerations and that these should prevail over the interests of science and society throughout the whole duration of the study.

The Sponsor has developed and implemented a robust study protocol and agreed proposals for substantial protocol amendments where necessary. Furthermore, they have produced (and continue to develop) reports to funders, regulatory bodies and collaborators where necessary.

Additionally, the Sponsor has been responsible for research sample collection procedures and management.

The Sponsor has also led discussions about the study data and advised on the data analyses and interpretation prior to potential submissions or publications.

**Table 1: Participant Timeline:**

| **Visits**  **Assessments** | **Screening**  **10 weeks (+/- 2 weeks)** | **Week 0** | | | **W1**  **+/-1 day** | **W2**  **+/-1 day** | **W3**  **+/-1 day** | **W5**  **+/-2 days** | **W8**  **+/-2 days** | **W16**  **+/-3 days** | **W21**  **+/-3 days** | **Safety**  **FUP**  **W52**  **+/- 2 weeks** | **Safety**  **FUP**  **W104**  **+/- 2 weeks** |
| --- | --- | --- | --- | --- | --- | --- | --- | --- | --- | --- | --- | --- | --- |
|  |  | **Day**  **- 1** | **Day 0** | **Day**  **1** |  |  |  |  |  |  |  |  |  |
| **Informed Consent** | **X** |  |  |  |  |  |  |  |  |  |  |  |  |
| **Inclusion/Exclusion Criteria Review^[[1]](#endnote-1)^** | **X** | **X** |  |  |  |  |  |  |  |  |  |  |  |
| **Medical History (including demographic data)** | **X** |  |  |  |  |  |  |  |  |  |  |  |  |
| **Concomitant medication** | **X** | **X** | **X** | **X** | **X** | **X** | **X** | **X** | **X** | **X** | **X** |  |  |
| **Adverse Events** | **X** | **X** | **X** | **X** | **X** | **X** | **X** | **X** | **X** | **X** | **X** | **X** | **X** |
| **Doctor review including Physical Examination** | **X** | **X** | **X** | **X** | **X** | **X** | **X** | **X** | **X** | **X** | **X** | **X** | **X** |
| **Vital Signs** | **X** | **X** | **X** | **X** | **X** | **X** | **X** | **X** | **X** | **X** | **X** |  |  |
| **12- lead ECG** | **X** |  | **X** |  |  |  |  |  |  |  |  |  |  |
| **Colonoscopy** | **X** |  |  |  |  |  |  |  | **X** |  |  |  |  |
| **Biopsy (Ileum and Colon)** | **X** |  |  |  |  |  |  |  | **X** |  |  |  |  |
| **MRI or CT abdomen/pelvis or U/S small bowel** | **X** |  |  |  |  |  |  |  |  |  |  |  |  |
| **Screening TB (chest x-ray and IGRA blood test)** | **X** |  |  |  |  |  |  |  |  |  |  |  |  |
| **Leukapheresis for TR004 manufacturing** | **X** |  |  |  |  |  |  |  |  |  |  |  |  |
| **Clinical Blood Tests** | **X** | **X** | **X** | **X** | **X** | **X** | **X** | **X** | **X** | **X** | **X** | **X** | **X** |
| **Blood samples for Translational Research** | **X** | **X** |  | **X** | **X** | **X** | **X** | **X** | **X** | **X** | **X** |  |  |
| **Serum Pregnancy Test** | **X** | **X** |  |  |  |  |  |  | **X** |  |  |  |  |
| **Stool samples collection** | **X** | **X** |  |  | **X** | **X** | **X** | **X** | **X** | **X** |  |  |  |
| **QoL questionnaire** |  | **X** |  |  |  |  |  | **X** |  | **X** |  |  |  |
| **Dispense patient diary** | **X** |  |  | **X** | **X** | **X** | **X** | **X** | **X** | **X** |  |  |  |
| **TR004 Dosing** |  | **X** |  |  |  |  |  |  |  |  |  |  |  |
| **Participant Experience Assessment** |  |  |  |  |  |  |  |  |  |  | **X** |  |  |

**Table 2: Timing of blood sampling for immune cell phenotyping and cytokine analysis:**

| **Sample Number** | 1 | 2 | 3 | 4 | 5 | 6 | 7 | 8 | 9 |
| --- | --- | --- | --- | --- | --- | --- | --- | --- | --- |
| **Trial Visit** | SCR | W0 **(D-1, D1)** | W1 | W2 | W3 | W5 | W8 | W16 | W21 |

1. [↑](#endnote-ref-1)
