# Supplementary material for: Protocol for a first-in-human feasibility study of T regulatory cells (TR004) for inflammatory bowel disease using (ex vivo) Treg expansion (TRIBUTE)
Source: BMJ Open. 2025 Jan 23;15(1):e092733. doi: 10.1136/bmjopen-2024-092733 (PMC11759877; doi:10.1136/bmjopen-2024-092733)
Supplement: online supplemental file 2 [file bmjopen-15-1-s002.pdf]

KING'S College LONDON

NHS Guy's and St Thomas' NHS Foundation Trust

INFORMED CONSENT FORM

A first in human feasibility study of T regulatory cells (TR004) for inflammatory bowel disease using (ex vivo) Treg expansion

The TRIBUTE Feasibility Study

Chief Investigator: **Professor Peter Irving**

Participant Identification Number:

Please initial box

1. I confirm that I have received a personal copy of, and have read and understood, the Patient Information Sheet dated ____________ (Version ___) for the above study and have had the opportunity to ask questions and discuss the study. I have been given a copy of the Patient Information Sheet to keep. ☐

2. I understand that my participation is voluntary and that I am free to withdraw at any time without giving any reason, without my medical care or legal rights being affected. ☐

3. I understand that sections of any of my medical notes and data collected during the study may be looked at by responsible individuals involved in this study, or by regulatory authorities, Guy's and St Thomas' NHS Foundation Trust, and the research and development department where it is relevant to my taking part in this research. I give permission for these individuals to have access to my records. I understand that my personal data will be processed and stored securely in compliance with the Data Protection Act 2018 and the General Data Protection Regulation. I understand that the data collected during the study may be sent outside of the UK and EEA. ☐

4. I agree to my GP being informed of my participation in this study. ☐

5. I consent to my donated white blood cells, obtained by leukapheresis, to being used for the manufacture of the cell therapy medicinal product. ☐

6. I understand that my donated white blood cells will be identified by my first and last name, date of birth, hospital number and study code number. ☐

7. I consent to being tested for HIV, Hepatitis B, Hepatitis C, HTLV and syphilis. ☐

8. I agree to the collection, storage and analysis of blood, stool and tissue samples. I understand that this research may involve laboratories outside of the UK and EEA, including commercial partners. ☐

9. I agree to use an effective method of contraception whilst taking part in the trial, as stated in the patient information sheet. ☐

10. I agree to take part in the above study. ☐

OPTIONAL: I understand that the information collected about me will be used to support other research in the future, and may be shared anonymously with other researchers. ☐

OPTIONAL: I agree to my samples being stored for use in future ethically-approved research studies, including samples of the Treg immunotherapy manufactured using my donated white blood cells. I agree to the collection, processing, storage and analysis of genetic material found in blood cells, stool and tissue samples. ☐

OPTIONAL: I would like to receive a summarised version of the study results following the end of the study ☐

Name of patient	Date	Signature
Name of Investigator	Date	Signature

When completed: 1 copy for patient; 1 copy for medical notes; 1 (**original**) to be kept in the Investigator Site File.
