# Supplementary material for: Protocol for a first-in-human feasibility study of T regulatory cells (TR004) for inflammatory bowel disease using (ex vivo) Treg expansion (TRIBUTE)
Source: BMJ Open. 2025 Jan 23;15(1):e092733. doi: 10.1136/bmjopen-2024-092733 (PMC11759877; doi:10.1136/bmjopen-2024-092733)
Supplement: Supplementary file 3 [file bmjopen-2024-092733.reviewer_comments.pdf]

PEER REVIEW HISTORY

BMJ Open publishes all reviews undertaken for accepted manuscripts. Reviewers are asked to complete a checklist review form and are provided with free text boxes to elaborate on their assessment. These free text comments are reproduced below.

ARTICLE DETAILS

Title (Provisional)

Protocol for a first in human feasibility study of T regulatory cells (TR004) for Inflammatory Bowel disease using (ex vivo) Treg expansion (TRIBUTE).

Authors

Rodger, Beverley; Clough, Jennifer; Vasconcelos, Joana; Canavan, James B; Macallan, D; Prevost, A Toby; Lord, Graham M; Irving, Peter

---

VERSION 1 - REVIEW

**Reviewer**	**1**
**Name**	**Kurada, Satya**
**Affiliation**	**Indiana University School of Medicine**
**Date**	**08-Sep-2024**
**COI**	**None**

Interesting pilot study to see effect of ex vivo expansion of autologousT regs and reinfusion.

VERSION 1 - AUTHOR RESPONSE

Dear BMJ Open Editorial Team,

Thank you to the editor and reviewers for their helpful comments and suggestions.
